# Social-Emotional Profile of Children with and without Learning Disabilities: The Relationships with Perceived Loneliness, Self-Efficacy and Well-Being

**DOI:** 10.3390/ijerph17207358

**Published:** 2020-10-09

**Authors:** Tali Heiman, Dorit Olenik-Shemesh

**Affiliations:** Department of Education and Psychology, The Open University of Israel, 1 University Road, Ra’anana 4353701, Israel; doritol@openu.ac.il

**Keywords:** children, social support, loneliness, self-efficacy, well-being

## Abstract

The current study examined whether perceived social support mediated the effects of loneliness and self-efficacy on well-being among students with or without a learning disability (LD). Participants included 834 elementary, middle, and high school students from Israel (29.6% students with LDs) who completed self-report questionnaires. The results of structural equation modeling indicate that social support mediates the indirect effects of age, gender, loneliness and self-efficacy on well-being. In addition, the results show differences between groups, as non-LD girls noted a higher self-efficacy and well-being than boys, and well-being had indirect effects in the non-LD group than in the LD group. These results indicate students with LDs have a unique social-emotional profile that affects their well-being. The study highlights the importance of enhancing self-efficacy and reducing loneliness in order to increase social support, thus predicting positive well-being. Effective and practical educational programs are needed for both groups across age and gender.

## 1. Introduction

Various studies discuss the social, emotional, and personal characteristics of individuals with a learning disability (LD), including their ongoing problems in establishing and maintaining mutual interpersonal relationships with peers [1]. Studies often report poor social skills, low peer acceptance, higher feelings of loneliness, and lower self-perception among LD students compared to their non-LD peers [2]. Only few studies have examined their well-being, and the unique relationships between social support, loneliness, self-efficacy, given the full-inclusion educational programs of students with LDs.

Although there are extensions on previous knowledge on the social and emotional characteristics of adolescents, there are no studies on the mediating effects of social support on students with and without LDs. The present study intends to fill the gaps in the literature and understand whether different aspects such as loneliness and social-emotional perceived self-efficacy might affect students’ well-being, and whether the contribution of social support to students with or without LDs attending mainstream classes makes a difference.

In general, students diagnosed with LDs are estimated to be between 5% and 15% of the population and might fail more often in courses than the general education students [3]. In Israel, since 1998, and with the recent revision of the Special Education Law (2018) prioritizing inclusion in in mainstream schools for students with special needs, the Ministry of Education has intensively supported the policy of inclusion. To date, most of the students diagnosed with LDs are integrated in mainstream educational programs from elementary schools [4].

### 1.1. Social Support

Social support is conceptualized as a protective factor that contributes to the individual’s successful adjustment in society, and is positively related to self-esteem [5] and well-being [6]. Lower social support, lack of close friends, unsecured connections with peers, or social rejection can evoke feelings of anger, unhappiness, and low self-esteem and decrease perceived well-being [7].

The challenges of students with LDs in the process of creating and maintaining social support are often a product of low social awareness and underdeveloped social skills and difficulties in keeping up in conversations with peers [8]. It was found that students with LDs reported low social support and low self-perception [9], whereas female students reported higher scores compared to male students. Within a longitudinal study regarding the predictors of success of individuals with LDs [10], all the participants referred to the effective assistance and support they had received from significant people in their lives, such as family members, teachers, and friends. The findings highlighted the important role of peers and family in offering social support, helping with routine tasks and/or skills, providing encouragement, and reducing stress among individuals with LDs.

### 1.2. Loneliness

Studies examining social relationships among students with LDs have indicated poorer relationships with their peers and frequent deficit in their social skills compared to typically non-LD students [11]. Prior research has revealed that individuals with LDs are at higher risk for feelings of loneliness from kindergarten through high school [12], and, among 716 adolescents with and without LDs, that the severity of the LD predicted stronger feelings of loneliness [2]. Within a recent study [13] it was found that adolescents with attention-deficit disorder (ADHD) reported significantly greater feelings of loneliness compared to their non-ADHD peers.

As for gender findings, inconsistent gender differences were noted regarding feelings of loneliness. Previous research noted that girls and boys might express feelings of rejection and loneliness differently across developmental stages—during childhood and adolescence [14]—and other studies reported higher feelings of loneliness among boys than among girls [15,16], while another study found that girls reported higher levels of loneliness than boys [17]. Furthermore, examining loneliness among youth revealed correlations with a lower self-efficacy and perceptions of self-competence, and that loneliness had a direct negative effect on adjustment and also negatively affects adjustment by activating a negative coping style [18].

### 1.3. Self-Efficacy

Perceived self-efficacy is a person’s belief of his or her ability to organize and successfully accomplish his or her behavior(s) that lead(s) to their desired results [19,20]. According to Bandura, factors such as anxiety and perceived self-efficacy affect the person’s behavior but are actually the product of feeling capable, influenced by previous experiences, background factors, and gender. Previous studies reveal that a strong sense of self-efficacy contributes to students’ growth and academic development. When students with LDs compare their performance with their peers without LDs, they tend to consider themselves as less valued and less skilled. Students with LDs also report lower academic self-efficacy and lower academic competence. Even when the academic performance of students with LDs is similar to that of typically achieving peers, their global self-perceptions continue to reflect lower self-efficacy and ongoing emotional distress [8]. Other research indicates that a low self-efficacy among children with LDs may be negatively influenced by classroom isolation, by the difficulties of dealing with school demands, and by repeated experiences of school failure [21], and noted that students with LDs tend to have lower self-esteem, self-acceptance, and self-efficacy [22]. Another study [23] suggested that as students with LDs experience fewer successes compared to their non-disabled peers, which often leads them to think they are failures, the repeated failures contribute to their low perceived self-efficacy.

Similar findings noted that students with LDs have low self-esteem, low self-acceptance, and low self-efficacy regarding their academic and social abilities [24,25]. Findings on adolescents with LDs reveal that they report higher levels of loneliness and lower levels of self-efficacy than typically achieving students. In addition, even with specific accommodations conducted for students with LDs in mainstream classes, students with LDs continue to consider themselves less academically competent, reporting lower academic achievements and lower academic self-efficacy [26]. Sporadic studies have examined gender differences related to self-efficacy, LDs and gender [27], revealing that, among high school students, LD status had an indirect influence on self-efficacy, but gender did not have direct or indirect influences on self-efficacy.

### 1.4. Subjective Well-Being

Subjective well-being (SWB) is a self-reported measure of well-being, typically obtained by a questionnaire developed by Diener in 1984 [28]. Definitions of SWB focus on how a person evaluates his/her own life, and comprises moods and emotions as well as an evaluations of one’s satisfaction with the general and specific areas of one’s life [29]. The conceptualization of SWB focuses on the quality of the individuals’ lives and includes both emotional feelings (positive or negative experience), and cognitive evaluations of life satisfaction. It can be viewed as an individual’s self-assessment of his or her life. Previous studies on SWB and gender differences among 300 youths found a positive correlation with self-esteem, as men scored higher in self-esteem. In addition, findings show that low self-esteem was associated with low levels of SWB, and high levels of self-esteem may predict high levels of SWB among emerging adults, for both women and men [30]. Other studies show that females reported poorer well-being indicators than males, and that females scored higher than males on the socio-emotional aspects and social connection, while males scored higher than females on self-efficacy [31]. Few studies examined perceived SWB focusing on students with LD. As such, boys with LDs reported the lowest perceived well-being and the highest levels of loneliness compared to girls with LDs and compared to non-LD students. Girls in the LD group reported the lowest levels of loneliness and a higher feeling of well-being than the boys with LDs [8].

### 1.5. The Current Study

Based on previous literature indicating that students with LDs have social and emotional problems, are at a greater risk of experiencing loneliness than students without LDs [1], exhibit a lower self-concept, and need more social support from family members and friends [2] than children without LDs, in the present study, we intend to better understand the relationships between these variables. The purpose of this study are: (a) to find whether students with and without LDs differed in the level of social support, loneliness, self-efficacy, and well-being; (b) to examine the effect of loneliness and self-efficacy on well-being mediated by social support; (c) to examine whether gender and age for each group of students differed in the level of social support, loneliness, self-efficacy, and well-being. Furthermore, the study aims were to understand whether the various factors might affect students’ well-being, and whether the impact differs between students with and without LDs. Because few studies have examined the unique effects given the full-inclusion educational programs for students with and without LDs, examining the relationships of personal and psychological measures are important. This study addressed the following research predictions:

1. We hypothesized that students with LDs would report lower social support, self-efficacy, and well-being, and higher loneliness than non-LD students.
2. We hypothesized that higher social support will have mediated the effects of loneliness and perceived self-efficacy on well-being for each group of participants (with and without LD).
3. Gender differences would occur across student groups: girls would report lower social support and lower well-being and higher loneliness than boys, compared to non-LD students.

## 2. Method

### 2.1. Participants

The current study included 834 school students, of which 70.4% (*n* = 587) were non-LD students and the rest were diagnosed with an LD (29.6%, *n* = 247). The participants were recruited from three types of schools: three elementary schools (354 students, 42.4%, age range = 10–12), three middle schools (307 students, 36.8%, age range = 13–15), and two high schools (173 students, 20.7%, age range = 16–17), with families of diverse socioeconomic backgrounds.

In the first two school types, three classes were sampled, whereas in the last, two classes were sampled. Students’ ages ranged from 10 to 17. The ratio of LD students to non-LD students was 32.2% in the high school, 30.3% in the middle school and 22.9% in the elementary school, χ_(2)_^2^ = 19.1, *p* < 0.05. Of the total sample, 50.6% were girls and 49.4% were boys. As in the learning disabilities group, this ratio was slightly larger for girls (52.8%) in the non-LD group and switched in the LD group (45.3%). This difference in the frequencies was significant according to a chi-squared test, χ_(1)_^2^ = 3.87, *p* = 0.049.

The inclusion/exclusion criteria for students with LDs were obtained by an educational counselor. To be identified as having an LD, the students needed to be diagnosed by an authorized educational psychologist or by a clinical psychologist specializing in learning disabilities. Students with LDs needed to pass a battery of tests, including a psycho-didactic diagnosis, which is a combined didactic and psychological diagnosis, a comprehensive didactic assessment of learning functions. The diagnostic assessments included instruments such as the Wechsler Intelligence Scale for Children, Kaufman Assessment Battery for Children, and the Hebrew adaptation of the Rey-Auditory Verbal Learning Test. All the students with LDs had an average IQ level (ranging from 85 to 115). Due to confidentiality law, no data were available regarding individual children’s psychoeducational evaluation. Students with LDs must demonstrate significant difficulties in at least one of these academic skills: reading, writing, and/or mathematics, compared to the typical peers, and to obtain substantially lower achievements scores on standardized psychology-educational tests, including reading, writing, or mathematics than expected for his/her age group.

According to Israeli Ministry of Educational policy, these students can receive special assistance by inclusive teachers during school hours, focusing on their specific needs. In the present study, researchers met the school counselor in each school. The school counselor identified the students diagnosed with LDs from the schools’ records. All the students identified with an LD had academic difficulties. The distribution of the 247 students’ impairments was as followed: reading difficulties (*n* = 75, 30%), writing difficulties (*n* = 56, 22%), combined writing and reading difficulties (*n* = 93, 38%), calculation difficulties (*n* = 14, 6%), and combined writing, reading and calculation difficulties (*n* = 10, 4%). The economic nurturing index measured students’ socio-economic background: 43.8% high, 33.6% mid-level, and 22.7% low. This ratio differed in middle school, where only 36.5% of the students were at the high level, χ_(4)_^2^ = 64.95, *p* < 0.001, whereas high school and elementary school students came from similar backgrounds regarding high versus other nurturing levels, χ_(1)_^2^ = 0.15, *p* = 0.70. Overall, the heterogeneity of the sample calls for a further control of age and gender in the final model.

### 2.2. Measures

Multidimensional scale questionnaire for perceived social support [32]: This questionnaire contains 12 items describing the participant’s current perception of the availability of social support from family, friends, or some other close, significant individual. Scale items are divided into three subscales and refer to support from (a) family, (b) friends, and (c) a close friend (e.g., “my family tries to help me,” “I can talk about my problems with my friend,” and “I have a close person that I can trust”). Participants responded on a 7-point Likert scale (1 = extremely unsuitable, 7 = extremely suitable), with higher scores indicating greater perceived social support. The reliability of Cronbach’s alpha for the entire questionnaire was 0.95.

Loneliness questionnaire [33]: The loneliness scale is a self-report measure that assesses an individuals’ perceived loneliness. The measure consists of 16 items that are scored on a 5-point scale, with higher scores indicating a high sense of loneliness. The questionnaire has two subscales (social loneliness and emotional loneliness), but in the analysis of the present study, we used the total scale (e.g., “I have many friends in my class,” and “I feel alone at school”). The reliability of Cronbach’s alpha for the entire questionnaire was 0.90.

Self-efficacy [34]: The entire scale includes social, emotional and academic dimensions of perceived self-efficacy. In the present study, we used only items assessing students’ views of their social and emotional perceived self-efficacy. The measure consists of 13 items that are scored on a 5-point scale, (1 = not at all to 5 = very well), with higher scores reflecting a higher sense of self-efficacy. The subscale of social self-efficacy has five items (e.g., “To what degree are you able to connect with other students?”). The emotional self-efficacy subscale is calculated as an average of eight items examining the respondents’ ability to monitor their feelings (e.g., “To what degree do you manage to calm yourself down when you are afraid?” “To what degree can you cheer yourself up when you feel depressed?”); reliability for the entire questionnaire alpha for the current study was 0.85.

Subjective well-being [35]: The scale was designed to measure global judgments of satisfaction with one’s life and aims to examine the respondent’s life as a whole [36]. The scale contains five items designed to measure global cognitive judgments of satisfaction with one’s life (e.g., “I am very satisfied with my life” and “If I could live my life again, I would change very little.”), on a 7-point scale (1 = strongly disagree, 7 = definitely agree). A high score represents a person with a higher perceived sense of well-being. Cronbach’s alpha of the overall scale of the present study was 0.86.

### 2.3. Procedure

The study was conducted in accordance with the Declaration of Helsinki, and the protocol was approved by the Ethics Committee of the Ministry of Education (Project 10059 identification code).

After receiving approval from the Israeli Ministry of Education, the researcher contacted the schools’ principals to receive their acceptance. A short email explaining the purpose of the study and guaranteeing the anonymity of the participants was sent to the school principals and to the parents of the students. The classes were randomly chosen by the school counselors. All the classes were considered mainstream classes, with average typical students.

Five parents refused to let their child to complete the research questionnaires. We excluded these students from the sample. The final sample included 834 students who completed the questionnaires in their classroom within one hour. The information regarding students’ specific disability were reported from school records. In order to keep the students’ anonymous, a specific mark was signed on the questionnaires of the LD students.

### 2.4. Data Analyses

**Figure 1:**
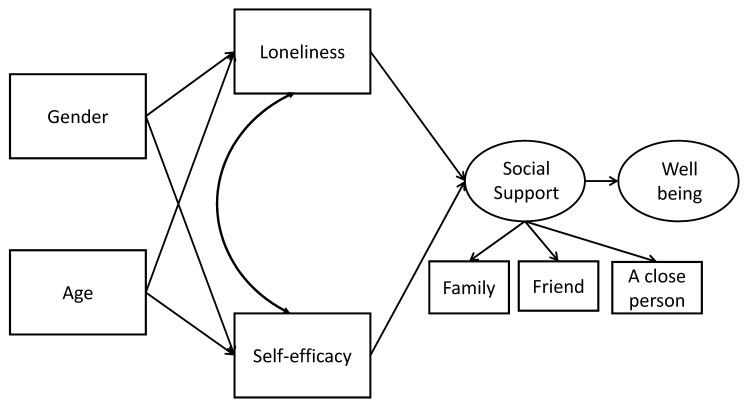
The conceptual hypothesis-testing model within a structural equation. Note: rectangles for observed variables, and ellipses for latent factors.

In this study, we adopted a structural equation modeling (SEM) approach with observed and latent factors for hypothesis testing. Conceptually, we present the model in Figure 1. The model combines exogenous background variables, personal status, social support, and well-being. Both social support and well-being are latent in the sense that an unobserved factor explains observed items that share a similar context as a set of simple linear regressions.

Within the research, a comparison will be examined between students with LDs and without LDs. The SEM approach allows for testing complex hypotheses simultaneously rather than independently. In addition, although the direct effects are estimated, alternative indirect effects may be assessed; that is, the effect of an independent variable on a dependent variable may be pronounced in the presence of a mediating variable. For example, social support mediates the effect of loneliness on well-being. In Figure 1, straight arrows represent paths from independent to dependent variables, and double-headed arches represent correlations between two variables. The model estimation is subject to the assumption that no correlations exist among independent variables, yet modifications to the model improve the fit if these correlations truly exist [37].

Before analysis, we examined the data for missing values and imputed missing data using a maximum likelihood estimation [38]. We then tested the mediational model through SEM using SPSS AMOS 25.0 (IBM Corporation, Armonk, NY, USA) with a maximum likelihood estimation to evaluate the model fit and to examine the direct and indirect relationship between students’ measures. We used three goodness-of-fit indices to assess the model fit: chi-square test, comparative fit index (CFI), and root mean square error of approximation (RMSEA). A good model fit is demonstrated when the model chi-square value is not significant, the CFI value is 0.95 or greater, and the RMSEA value is 0.06 or less [39]. We used an alpha level of *p* < 0.05 to determine statistical significance.

To test for mediation, we used the joint significance test of indirect paths from the predictor (loneliness and self-efficacy) to the mediator (social support), and from the mediator to the outcome (well-being). We used bias-corrected bootstrapping in which indirect effects are estimated from multiple resampling from the dataset.

## 3. Results

### 3.1. Preliminary Analysis

In order to examine the first hypothesis, that students with LDs would report lower social support, self-efficacy, and well-being, and higher loneliness than non-LD students, a preliminary analysis was conducted, comparing indicators between the two groups within a multivariate analysis of variance (MANOVA) test.

Table 1 shows self-efficacy and well-being indicators were higher among the non-LD students than among the LD students, *F* (1832) = 5.18, *p* < 0.05; *F* (1832) = 5.83, *p* < 0.05, respectively. The overall difference was insignificant—Wilks’ Λ = 0.986, *p* = 0.079—and complementary analyses for the social support items and the well-being items resulted in similar insignificant values—Wilks’ Λ = 1.00, *p* = 0.966; Wilks’ Λ = 0.991, *p* = 0.221, respectively.

As presented in Table 2, Cronbach’s alpha exceeded 0.85 for all the measures. Moderate correlations were found across these indicators both in the non-LD and the LD groups (see Table 2 and reliability indices).

The significant correlations were between 0.26 and 0.55, except for higher correlations between sources of social support (family support, friend support, close-person support, r = 0.58–0.78). Thus, we built a latent factor for these items, as well as a well-being factor. The composite reliability of the factors was high (social support: CR = 0.85, *p* < 0.001; well-being: CR = 0.93, *p* < 0.001). Furthermore, a multiple group comparison of the measurement model for the two latent factors—social support and well-being—revealed that factor structures were similar for both groups of students (LD and non-LD groups), as shown in Table 3.

In order to detect different associations between factors, we ran separate models simultaneously within the multiple group comparison framework. In other words, regression coefficients across groups were unconstrained, whereas loadings were constrained equally. All factor loadings were above 0.65.

### 3.2. Modeling Results

**Figure 2:**
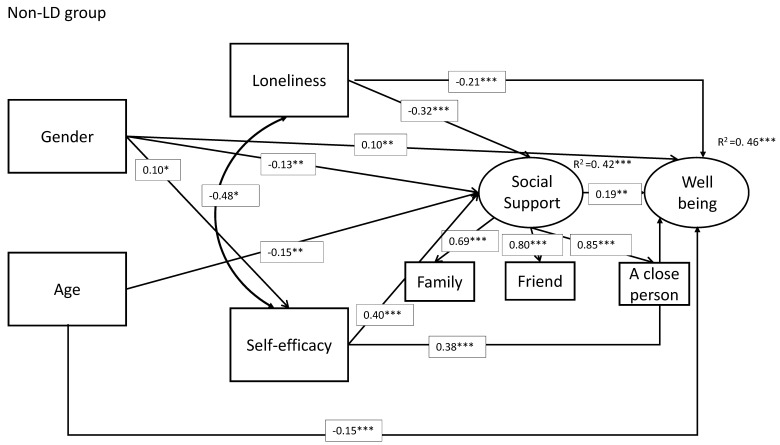
The model within a structural equation framework for non-LD group. *** *p* < 0.001; ** *p* < 0.01; * *p* < 0.05.

**Figure 3:**
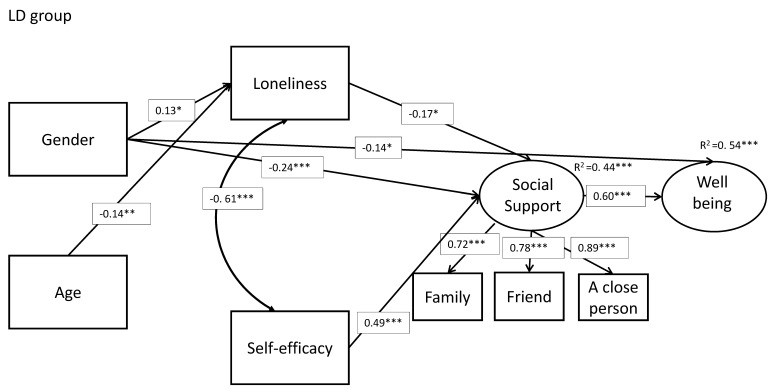
The model within a structural equation framework for learning disability (LD) group. *** *p* < 0.001; ** *p* < 0.01; * *p* < 0.05.

We hypothesized (hypothesis 2) that higher social support will have mediated the effects of loneliness and perceived self-efficacy on well-being for each group of participants (students with and without LD). Model results are displayed in Table 4 (unstandardized estimates) and Figure 2 and Figure 3 (standardized estimates).

Findings show that loneliness negatively affected the support and the well-being outcomes (b = −0.04, *p* < 0.001; b = −0.03, *p* < 0.001, respectively), whereas self-efficacy had the opposite effect (b = 0.72, *p* < 0.001; b = 0.72, *p* < 0.001, respectively). Social support was positively associated with well-being.

Results regarding students with LDs revealed that social support mediated several effects on well-being (age: indirect = 0.02, *p* < 0.05; loneliness: indirect = −0.007, *p* < 0.01; self-efficacy: indirect = 0.14, *p* < 0.05; gender: indirect = −0.06, *p* < 0.05). In addition, it was found that social support mediated self-efficacy and gender effects on well-being (indirect = 0.56, *p* < 0.001; indirect = −0.43, *p* < 0.01, respectively). Indirect effects were found to a higher extent among the non-LD students (see Table 5). These indirect effects mean the mediating effect of the social-support factor can explain the effect of independent variables on well-being. In addition, self-efficacy mediated the gender effect on well-being (indirect = 0.11, *p* < 0.05). Table 5 presented Indirect effects by group.

Hypothesis 3 focused on gender differences. Assuming that across student groups, girls would report lower social support and lower well-being, and higher loneliness than boys, and compared to non-LD students.

Findings regarding gender and age revealed a mediation effect as girls having higher self-efficacy, and higher self-efficacy is positively associated with higher well-being. Girls in the non-LD group showed higher self-efficacy and well-being (b = 0.15, *p* < *0*.05; b = 0.27, *p* < 0.01, respectively), but they reported lower support than boys (b = −0.33, *p* < 0.01). Older students reported higher support but lower well-being (b = 0.09, *p* < 0.01; b = −0.10, *p* < 0.001, respectively).

Among the LD students’ group, gender differences reveal that girls felt lonelier than boys (b = 3.08, *p* < 0.05). Girls reported lower support but higher well-being than boys (b = −0.65, *p* < 0.001; b = 0.41, *p* < 0.05, respectively), and older students reported lower loneliness (b = −0.75, *p* < 0.05). Self-efficacy was positively associated with social support (b = 0.85, *p* < 0.001), such that students with higher self-efficacy also experienced higher social support. The social-support experience was associated with higher well-being (b = 0.67, *p* < 0.001).

To conclude, results show that girls with LDs reported higher loneliness and lower social support than girls without LD. Older boys with LDs reported higher loneliness than girls with LD; subjective well-being was significantly correlated with age, higher self-efficacy, and lower loneliness among the non-LD group. In both groups, similar correlations were obtained in between well-being and gender (girls with higher well-being), and social support positively mediated perceived subjective well-being.

## 4. Discussion

The purpose of the present study was to examine the mediating effect of social support in relation to age, gender, loneliness, self-efficacy, on perceived well-being, among students with and without LDs attending mainstream classes.

The research results show that loneliness negatively affected the social support and SWB, and social support was positively associated with well-being. What is novel in these findings is that social self-efficacy is directly related to social support as a mediator of well-being in both groups. Regarding the group of students with LDs, the findings revealed that social support mediated SWB, and that social support mediated the self-efficacy and gender effects on SWB. These effects were direct and indirect.

In addition, the present study adds to our understanding of the unique contribution of age and gender on the internal (e.g., self-efficacy, loneliness) and external (social support) predictors, as well as to better understand the relationships between the various measures, and focuses on the differences and similarities between students with and without LDs.

Regarding the hypothesis, focusing on the differences and similarities between students with and without LD, as was previously found [40], the findings revealed that students with LDs reported a lower perceived social support compared to peers without LDs. As previously suggested [41], adolescents with LDs may experience poorer parent, classmate, and friend support. Furthermore, as expected, and similar to other previous results [42], in this study, students with LDs reported higher levels of loneliness and lower well-being than peers without LDs, and students with LDs frequently experience significant lower self-efficacy than peers.

Focusing on the mediation analysis for non-LD students, the findings revealed that many measures were related to well-being, such as age, gender, loneliness, and high perceived self-efficacy, directly mediated by social support. Among the LD group, all the measures are indirectly related to well-being, mediated by social support. As such, contrary to the non-LD group, gender and age did not affect self-efficacy, whereas among the non-LD group, girls had a higher perceived self-efficacy than boys. The results highlight the central role of social support and feelings of loneliness for LD students along the three stages of development (elementary, middle, and high school).

The findings also show that feelings of loneliness and perceived self-efficacy are significantly related to social support for both groups of students, yet age and gender are related to loneliness only among the LD group. Interestingly, perceived self-efficacy was directly related to well-being among non-LD students, but not among the LD students’ group. We can suggest the students with LDs have more concerns regarding their social support and loneliness. Consistent with previous studies [43] regarding students with LDs and perceived self-efficacy, the present findings revealed that students with LDs were more likely to possess a low perceived self-efficacy compared to students without LDs. The novel findings are that self-efficacy is directly related to social support, as a mediator of well-being in both groups. Higher self-efficacy appears to be positively related to social support and well-being. As such, developing and enhancing students’ self-efficacy may be necessary, because it has a significant effect on well-being. Such an education program may be a powerful approach to assist at-risk students with lower self-efficacy, lower social support, and lower well-being.

As was hypothesized, girls with LDs felt lonelier that boys with LDs; girls with LDs reported higher levels of loneliness, and reported lower social support, suggesting girls are more vulnerable than boys among the different age groups (elementary, middle and high school). This higher perceived well-being among girls, compared to boys, in both groups, might be due to the inclusion in mainstream classes, which enhanced the girls’ well-being.

These results are in line with the study hypotheses and are similar with some literature findings as adolescents’ boys reported lower emotional support from the people in their social networks compared to girls, and lower perceived parent support than girls [41]. In the present study, girls from both groups reported higher well-being compared to boys. The results are contrary to the study expectations, as girls reported higher levels of subjective perceived well-being compared to boys, and the same trend was found among girls with and without LDs. As a strong connection was found between social support and well-being, it might be useful to improve students’ social support in order to decrease loneliness and to increase their well-being. In addition, the involvement of sensory perception among individuals with LDs [44], as well as among individuals with hyposensitivity or hypersensitivity [45], reflect their unique sensory perception profile (as auditory, visual and phonological processing), which significantly co-occurs with emotional or behavioral problems. Therefore, further investigations are recommended to examine the unique sensory profiles and their functional impact on individuals with LD, and the interactions between these characteristic of LD students and their social-emotional characteristics

### 4.1. Limitations and Future Directions

The present study has certain limitations worth noting. First, we relied on schools’ general information, as specific description of the participants’ characteristics was confidential. Future studies should consider examining the diversity of the participants as related to the social and emotional variables. Second, we conducted the study during a limited period. Conducting longitudinal studies that examine these variables at the beginning and at the end of the academic year may be of interest. Therefore, the generalizability of the conclusions of the findings should be interpreted with precaution. In order to obtain a broader view of the perceived internal feelings (e.g., loneliness, self-efficacy, and well-being) and external social support through various development ages, future studies should cover a large sample from various geographical areas, using similar designs with a sample of individuals with various LDs (e.g., reading, writing, calculation disabilities) to confirm this relationship. In addition, as the LD group contains both students with generic learning difficulties and students with specific learning difficulties (e.g., dyslexia, dyscalculia, etc.), it would be interesting in future studies to conduct separate analyses for these groups; it might also be interesting to integrate multiple respondents, such as parents, teachers, or peers, in order to obtain an additional perceptive of students’ social and personal skills.

Despite these limitations, findings from this study have important implications for research and practice, which add to those of previous studies showing that students with LDs appear to experience loneliness in different developmental periods, whereas this connection was not found among typical students.

The findings of the present study provide insights into the role of social support in the LD students. Past research has not examined the impact of social support on perceived well-being in LD, through perceived self-efficacy and loneliness, therefore further in-depth work is needed. This study has also provided additional evidence for the important role of social support, especially for students with LDs, studying in mainstream classrooms. Other research that clearly examines the nature of the relationship between these variables in various educational settings of students with LDs are needed.

### 4.2. Implications for Practice

The implications of these findings could be useful to the educational staff, counselors and school psychologists who are responsible for supporting the social, emotional, and behavioral needs of the students. Specifically, students with disabilities, from elementary to high school age need help to overcome their continuous and multiple social and emotional difficulties in order to develop effective life-long learning, well-being, and positive self-efficacy. As was previously suggested [42,43], the current findings also suggest the implementation of classroom interventions focused on increasing the social relationships of students with disabilities. We recommend that intervention programs be developed in order to enhance social support in students with LDs studying in mainstream classrooms. Ongoing programs, over several months, may have a significant longer-lasting effect on the students. These educational programs, should enhancing awareness to students with disabilities, should discuss the benefits and the difficulties of inclusion, through learning new social strategies, and motivate students for socially support and strengthening their peer circle, in order to decrease feelings of loneliness, raising perceived self-efficacy, and improve the probability of positive well-being. Future research could examine the effectiveness of such a program.

The educational team need to find ways to increase or to enhance the students’ sense of (social) self-efficacy, to implement more social programs in schools, in order to increase social interactions, mutual friendships and peer acceptance with peers with and without disabilities. Practically, it might be worthwhile to provide training and consultation to teachers regarding their work with students with LDs, and it might be challenging for teacher education programs to allocate time in their studies to address practical coping tools regarding social and emotional aspects, as increasing children’s personal self-efficacy and perceived well-being.

Teachers have an important role in reducing the possible negative social-emotional consequences, and engaging individual or systemic interventions programs in the classroom; therefore, it might be useful to provide specific training to help them respond to the students’ difficulties. Specific educational programs need to focus on what activities to implement in the class, and on how educators and other professional school support staff interact with the children at risk for social and emotional exclusion. Educational staff should promote social interventions related to friends’ support, to help reduce loneliness and increase the perceived lower self-efficacy (as was found among students with LDs compared to non-LD peers), by supporting the students, helping them to learn appropriate social skills, and helping them to decrease loneliness, in order to strengthen a higher and more positive well-being. In conclusion, this study makes a clear contribution to the literature by identifying the unique differences and similarities between students with and without LDs. The findings highlight the need to improve LD students’ well-being, perceived self-efficacy, and levels of social support.

## 5. Conclusions

The findings may encourage additional research of students with LDs included in mainstream classes, in order to deeply investigate the outcomes of the inclusion on their perceived social support, loneliness, self-efficacy on well-being.

The findings revealed that the perceived social support mediated the effects of loneliness and self-efficacy on well-being among students with or without a learning disability. Students with LDs have a unique social-emotional profile that affects their well-being. For example, students with LDs reported lower social self-efficacy and lower SWB and feelings of loneliness from elementary to high school. Additionally, age and gender are related to loneliness only among the LD group.

In addition, differences between groups show that the non-LD female noted a higher self-efficacy and well-being than males, and well-being had indirect effects in the non-LD group than in the LD group. Gaining a further understanding of the unique social-emotional characteristics of students with LDs may improve their feelings and interactions with peers, and consequently, contribute to their well-being.

## Figures and Tables

**Table 1:** Multivariate analysis of variance for observed indicators.

	Non-LD Group (*n* = 587)		LD Group (*n* = 247)			
	*M*	*SD*	*M*	*SD*	*F*(1832)	*η* ^2^ *_p_*
Loneliness	27.71	11.87	28.26	12.27	0.37	0.00
Self-efficacy	3.72	0.77	3.58	0.82	5.18 *	0.006
Family support	5.81	1.91	5.91	1.69	0.58	0.001
Friend support	5.30	2.05	5.37	1.97	0.21	0.00
Close person support	5.70	1.91	5.75	1.81	0.10	0.00
Well-being	5.61	1.31	5.36	1.48	5.83 *	0.007

* *p* < 0.05; *η*^2^*_p_*—Eta Partial Squared.

**Table 2:**
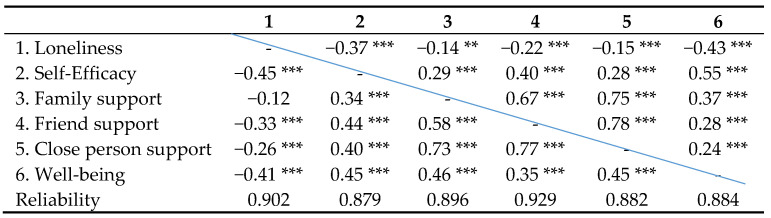
Correlations across research items.

Upper Triangular—Non-LD group; Lower Triangular—LD group. *** *p* < 0.001, ** *p* < 0.01.

**Table 3:** Multiple group comparison for latent factors.

Fit Parameter	χ^2^(df), *p*	CFI	TLI	RMSEA	SRMR
Model					
One Model	11.75(6), 0.068	0.998	0.995	0.034	0.009
M1: Configured	26.72(12), 0.009	0.995	0.987	0.054	0.014
M2: Metric	36.64(16), 0.001	0.992	0.984	0.060	0.037
M3: Scalar	40.36(20), 0.005	0.993	0.989	0.049	0.039
∆M2-M1	12.92(4), 0.012	0.003			
∆M3-M1	13.64(8), 0.092	0.002			
∆M3-M2	0.72(4), 0.095	0.001			

**Table 4:** Model estimates by group, unstandardized coefficients.

	Loneliness	Self-Efficacy	Support	Well-Being
**Non-LD group**				
Gender	0.47 (0.94)	0.15 * (0.06)	−0.33 ** (0.11)	0.27 ** (0.09)
Age	0.03 (0.24)	−0.01 (0.2)	0.09 ** (0.03)	−0.10 *** (0.02)
Loneliness			−0.04 *** (0.01)	−0.03 *** (0.01)
Self-efficacy			0.72 *** (0.12)	0.72 *** (0.10)
Social support				0.20 ** (0.06)
R^2^	0.00 (0.00)	0.01 (0.01)	0.41 *** (0.05)	0.46 *** (0.04)
**LD group**				
Gender	3.08 * (1.42)	−0.01 (0.10)	−0.65 *** (0.16)	0.41 * (0.19)
Age	−0.75 * (0.34)	−0.01 (0.02)	0.05 (0.04)	−0.06 (0.04)
Loneliness			−0.02 (0.01)	−0.01 (0.01)
Self-efficacy			0.85 *** (0.18)	0.27 (0.20)
Social support				0.67 *** (0.14)
R^2^	0.04 * (0.02)	0.001 (0.01)	0.45 *** (0.06)	0.54 *** (0.06)

*** *p* < 0.001, ** *p* < 0.01, * *p* < 0.05; Model fit indices: CFI = 0.946; TLI = 0.918; χ^2^ = 260.98, df = 58, *p* < 0.001; RMSEA = 0.060.

**Table 5:** Indirect effects by group, unstandardized coefficients.

Independent	Mediator	Dependent	Independent → Mediator	Mediator → Dependent	Independent → Dependent	Indirect Effect	95% CI Indirect Effect
**Non-LD group**							
Age	Social support	Well-being	0.09 ** (0.03)	0.20 ** (0.06)	−0.10 *** (0.02)	0.02 * (0.01)	[0.01, 0.03]
Loneliness	Social support	Well-being	−0.04 *** (0.01)	0.20 ** (0.06)	−0.03 *** (0.01)	−0.007 ** (0.003)	[−0.01, −0.003]
Self-efficacy	Social support	Well-being	0.72 *** (0.12)	0.20 ** (0.06)	0.72 *** (0.10)	0.14 * (0.06)	[0.06, 0.28]
Gender	Self- efficacy	Well-being	0.15 * (0.06)	0.72 *** (0.10)	0.27 ** (0.09)	0.11 * (0.04)	[0.03, 0.20]
Gender	Social support	Well-being	−0.33 ** (0.11)	0.20 ** (0.06)	0.27 ** (0.09)	−0.06 * (0.03)	[−0.14, −0.02]
**LD group**							
Self-efficacy	Social support	Well-being	0.85 *** (0.18)	0.67 *** (0.14)	0.27 (0.20)	0.56 *** (0.15)	[0.32, 0.88]
Gender	Social support	Well-being	−0.65 *** (0.16)	0.67 *** (0.14)	−0.43 ** (0.14)	−0.43 ** (0.14)	[−0.73, −0.19]

*** *p* < 0.001, ** *p* < 0.01, * *p* < 0.05.
